# The Effect of Proton Pump Inhibitor Use on Survival of Patients With Colorectal Cancer: A Study of a Racially Diverse Population

**DOI:** 10.7759/cureus.38707

**Published:** 2023-05-08

**Authors:** Adham E Obeidat, Gabriel Monti, Horyun Choi, Jared Acoba

**Affiliations:** 1 Internal Medicine, University of Hawaii, Honolulu, USA; 2 Internal Medicine, Oregon Health & Science University, Portland, USA; 3 Internal Medicine, University of Hawaii Internal Medicine Residency Program, Honolulu, USA; 4 Hematology and Oncology, The Queen's Medical Center, Honolulu, USA

**Keywords:** hypergastrinemia, overall survival, mortality, colon cancer, proton pump inhibitor

## Abstract

Introduction

Proton pump inhibitor (PPI) use is increasing in the general population. Chronic PPI use can lead to hypergastrinemia, which has been purported to increase the risk of developing colorectal cancer (CRC). Several studies have failed to report any association between PPI use and the risk of CRC. However, little is known about the effect of PPI use on CRC survival. In this retrospective analysis, we studied the effect of PPI use on CRC survival in a racially diverse population.

Methods

Data were abstracted for 1050 consecutive patients diagnosed with CRC from January 2007 to December 2020. The Kaplan-Meier curve was created to study the effect of PPI exposure compared to no exposure on overall survival (OS). Univariate and multivariate analyses were performed to investigate predictors of survival.

Results

Complete data were available for 750 patients with CRC, 52.5% were males, 22.7% were Whites, 60.1% were Asians, and 17.2% were Pacific Islanders. A total of 25.6% of patients had a history of PPI use. Moreover, 79.2% had hypertension, 68.8% had hyperlipidemia, 38.0% had diabetes mellitus, and 30.2% had kidney disease.

There was no difference in median OS among PPI users compared to non-users, p value=0.4. Age, grade, and stage were predictors of inferior OS. No significant association was noticed with gender, race, comorbidities, or treatment with chemotherapy.

Conclusion

In this retrospective analysis of a racially diverse population of CRC patients, we found that PPI use was not associated with worse OS. Until high-quality prospective data are available, physicians should not stop PPIs that are clinically indicated.

## Introduction

Proton pump inhibitors (PPI) are used to manage various gastrointestinal diagnoses such as peptic ulcer disease and gastroesophageal reflux disease (GERD) [1,2]. The usage of PPI is increasing in the general population [2]. Chronic PPI use can lead to hypergastrinemia, which has been purported to increase the risk of colorectal cancer (CRC) development as serum gastrin can promote replication of both benign and malignant colonic epithelial cells [3-6]. Furthermore, animal studies have shown the progression of colon adenomas in the setting of hypergastrinemia [7]. On the other hand, pantoprazole has been shown in vitro to inhibit T-cell-originated protein kinase (TOPK), which is highly expressed in CRC cells and can promote tumorigenesis and progression, while another study showed similar anti-TOPK activity in vitro and in vivo with mice using ilaprazole [8,9].

Multiple observational studies and meta-analyses conducted across different populations have failed to report any association between long-term PPI and the risk of CRC [10-18]. However, one retrospective analysis studied the effect of PPI use on CRC survival and suggested a potential adverse effect of PPI use on the overall survival (OS) of CRC patients [19]. This study was limited by the small number of CRC patients using PPI. Moreover, the racial make-up of the study participants was not described, thus it is unknown if these results would be generalizable to a wider population. The primary objective of this study was to evaluate the impact of PPI use on survival among a large, racially diverse cohort of CRC patients.

An abstract of this study was presented as a poster at the American College of Gastroenterology 2021 Annual Scientific Meeting, Las Vegas, October 22-27, 2021.

A preprint of this study was published online on Research Square.

## Materials and methods

Patients and data collection

A retrospective analysis was performed on data gathered from the Queen’s Medical Center (QMC), Honolulu, Hawaii, Oncology Data Registry (ODR). QMC is the largest hospital in Hawaii and treats 40-50% of all colon cancer patients in the state. ODR was established in 1960 as part of the Hawaii Tumor Registry and has been contributing data to the Surveillance, Epidemiology, and End Results (SEER) program since 1973. All patients diagnosed with colorectal adenocarcinoma between January 1, 2007, and December 30, 2020, were eligible.

Institutional Review Board (IRB) approval was obtained from QMC for the conduct of this study; IRB approval number RA-2020-013. Data on patient demographics, clinicopathologic characteristics, and survival were collected from medical records. Race was self-reported by the patient and documented in the ODR. Patients were categorized into three racial groups: Asian (Korean, Chinese, Japanese, Filipino, Asian Indian (Indian and Pakistani), Southeast Asian (Thai, Vietnamese, Cambodian, and Laotian), and other Asian), Pacific Islander (Native Hawaiian, Samoan, Tongan, Micronesian, Marshallese, Fijian, Chamorro, and other Pacific Islander), and White. Patients of other races or unknown races only made up 1.2% of our study population and were excluded from the analysis.

Statistics

The primary objective was to study the potential effect of PPI use on OS among patients diagnosed and treated for CRC. Nonparametric descriptive statistics were used to evaluate characteristics of standard demographic, clinical, and tumor data. A two-sided p < 0.05 was considered statistically significant. OS was calculated by the Kaplan-Meier method and univariate comparisons between groups were carried out by using the log-rank test. Cox proportional hazards regression models for survival were built to obtain hazard ratios (HR) and 95% confidence intervals (CI) adjusting for age, gender, race, histologic grade, stage, surgery, chemotherapy, PPI use, and associated comorbidities. Statistical analyses and survival graphics were performed with R 4.0.3 (The R Foundation for Statistical Computing).

## Results

Demographic and clinical characteristics

**Figure 1:**
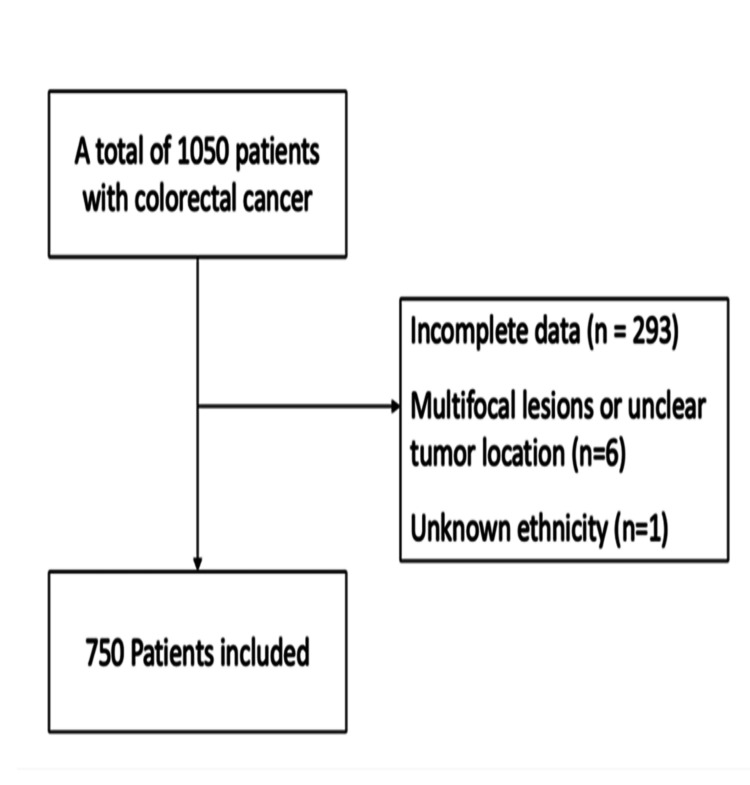
Flow chart of patients after applying inclusion and exclusion criteria.

In this study, a cohort of 1050 patients with CRC was identified, and 750 patients were included after excluding patients who did not have complete data (n=293) had multifocal lesions or unclear tumor location (n=6), and had unknown ethnicity (n=1) (Figure 1).

Table 1 shows details about the patient’s demographic and clinical characteristics (Table 1). There were 394 males (52.5%) and 356 females (47.4%). The median patient age was 66 (range, 28-100). We identified 192 patients (25%) who were using PPI, 71 of whom (37%) used PPI for over a year. There was no significant difference in race, tumor location, grade, and stage among patients with a history of PPI use compared to no use. There were more patients with hypertension (HTN), hyperlipidemia (HLD), diabetes mellitus (DM), and chronic kidney disease (CKD) among those with a history of PPI use. All patients received surgery and 48% (360) received chemotherapy.

**Table 1:** Baseline demographic and clinical characteristics of patients with colorectal cancer. BMI: body mass index; HTN: hypertension; HLD: hyperlipidemia; CKD: chronic kidney disease; PPI: proton pump inhibitor

Variable	No PPI use (n=558)	PPI use (n=192)	P-value
Age	66	67	0.314
Sex			0.286
Male	300 (53.8%)	94 (49.0%)	
Female	258 (46.2%)	98 (51.0%)	
Race			0.211
White	118 (21.1%)	52 (27.1%)	
Asian	340 (60.9%)	111 (57.8%)	
Native Hawaiian and other pacific islanders	100 (17.9%)	29 (15.1%)	
Tumor location			0.680
Right colon	163 (29.2%)	58 (30.2%)	
Left colon	184 (33.0%)	56 (29.2%)	
Transverse colon	43 (7.7%)	13 (6.8%)	
Rectum	168 (30.1%)	65 (33.9%)	
Lymph node involvement			0.900
No	309 (55.4%)	108 (56.2%)	
Yes	249 (44.6%)	84 (43.8%)	
Grade			0.774
Grade 1 + 2	469 (84.1%)	159 (82.8%)	
Grade 3 + 4	89 (15.9%)	33 (17.2%)	
Stage			0.214
Stage I	137 (24.6%)	40 (20.8%)	
Stage II	151 (27.1%)	67 (34.9%)	
Stage III	208 (37.3%)	67 (34.9%)	
Stage IV	62 (11.1%)	18 (9.4%)	
Surgery			
Yes	558 (100%)	192 (100%)	
Chemotherapy			0.656
No	287 (51.4%)	103 (53.6%)	
Yes	271 (48.6%)	89 (46.4%)	
BMI	25.8	25.6	0.880
HTN			< 0.001
No	236 (42.3%)	40 (20.8%)	
Yes	322 (57.7%)	152 (79.2%)	
HLD			<0.001
No	268 (48.0%)	60 (31.2%)	
Yes	290 (52.0%)	132 (68.8%)	
DM			<0.001
No	424 (76.0%)	119 (62.0%)	
Yes	134 (24.0%)	73 (38.0%)	
CKD			<0.001
No	509 (91.2%)	134 (69.8%)	
Yes	49 (8.8%)	58 (30.2%)	HLD:

OS in patients with PPI users compared to non-users

**Figure 2:**
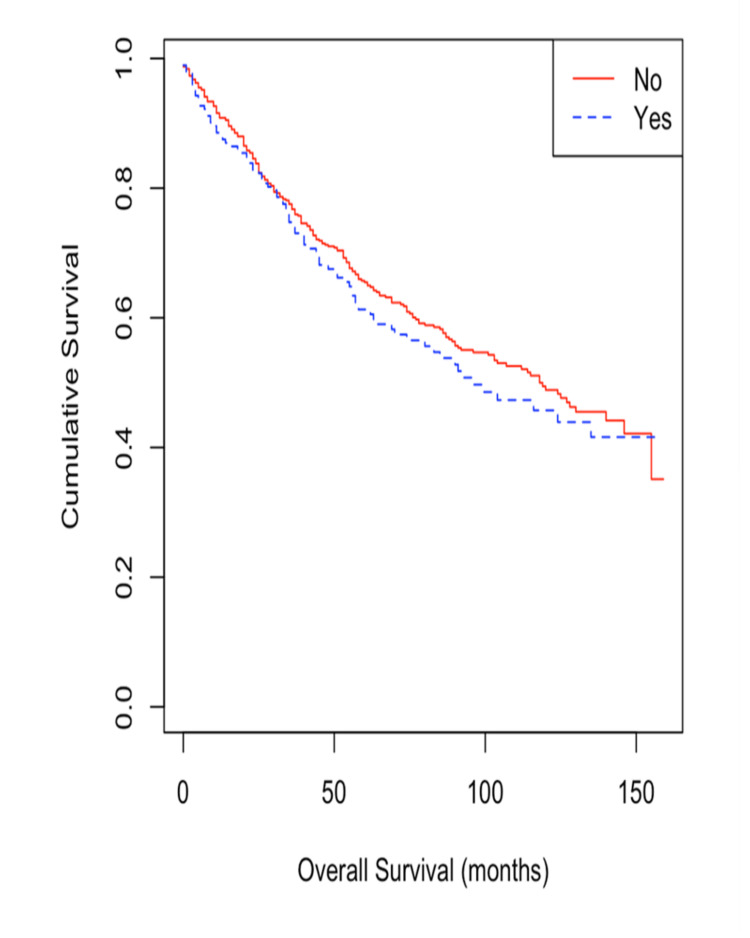
Kaplan-Meier survival curve for CRC patients with PPI exposure vs no exposure. CRC: colorectal cancer; PPI: proton pump inhibitor

The cohort was divided according to PPI use into two distinct groups and evaluated for OS using Kaplan-Meier curves. Patients with a history of PPI use demonstrated shorter median survival time compared with patients without a history of PPI use (96 months vs 118 months) (Figure 2), however, this difference was not significant (p-value=0.4). Even after adjustment for demographic factors, tumor characteristics, and comorbidities, the difference in median OS between PPI users and non-users remained non-significant (Table 2).

**Table 2:** Univariate and multivariate Cox regression model for potential predictors of overall survival in colorectal cancer patients. DM: diabetes mellitus; HTN: hypertension; HLD: hyperlipidemia; CKD: chronic kidney disease; PPI: proton pump inhibitor

	Univariate		Multivariate	
	HR (95% CI)	p-value	HR (95% CI)	p-value
Sex (1=men, 2=women)	0.889 (0.7142 to 1.107)	0.293		
Age (continuous)	1.035 (1.026 to 1.044)	< 0.001	1.039 (1.029 to 1.048)	< 0.001
Race (reference = white)				
Asian	0.947 (0.728 to 1.232)	0.684		
Native Hawaiian or Other Pacific Islander	0.937 (0.661 to 1.330)	0.716		
Grade (reference = G1-2)				
G3-4	1.765 (1.349 to 2.308)	< 0.001	1.471 (1.122 to 1.928)	0.008
Stage	1.713 (1.509 to 1.943)	< 0.001	2.134 (1.806 to 2.520)	< 0.001
Chemotherapy (reference = no)	0.995 (0.800 to 1.238)	0.965		
PPI	1.111 (0.869 to 1.420)	0.402	1.095 (0.846 to 1.418)	0.482
HTN	1.133 (0.902 to 1.424)	0.284		
HLD	0.913 (0.734 to 1.137)	0.416		
DM	1.028 (0.806 to 1.311)	0.822		
CKD	1.456 (1.098 to 1.931)	0.009	1.167 (0.867 to 1.572)	0.273

Predictors of OS in CRC patients

On both univariate and multivariate analysis, age, grade, and stage of the tumor were all negative predictors of OS. However, CKD was a negative predictor of OS on univariate analysis only. Conversely, gender, race, treatment with chemotherapy, HTN, HLD, and DM did not have a significant effect on OS (Table 2).

## Discussion

This study did not demonstrate an association between PPI use and median OS in patients with CRC. This is the second retrospective analysis conducted to examine this association, but unlike that conducted by Graham et al., our study featured considerably more patients (117 vs 192) who were using PPI [19]. While Graham et al. only reported about 9% of their patients were using a PPI, our study showed over 25% were exposed to a PPI, 37% of which used the PPI for over a year. This likely more closely represents the general population given the increased use of PPI [2]. In addition, fewer patients in our study had an advanced CRC stage, with around 47% having stage III or IV as compared to 62% of patients in the Graham et al. study. This difference is relevant, as a more advanced disease would significantly impact OS and may explain the observed association between PPI use and OS in Graham et al. study.

Our study showed no difference in demographic characteristics among patients who used PPI compared to non-users. Although there was a higher incidence of HTN, HLD, DM, and CKD among patients with a history of PPI use, there were no differences in histologic grade, stage, or location between the two groups. The absence of an association between PPI use and known negative prognostic factors supports our conclusion that PPI use does not impact OS among CRC patients.

Graham et al. did not describe the racial makeup of their study population, but it is likely that our study had a higher proportion of Asian patients given that they comprised 60% of the study population [19]. It has been widely noted that the incidence and mortality rates of CRC are lowest in Asians and Pacific Islanders in the United States, though more recent studies suggest that both the incidence and mortality of CRC are rising in Asia [20,21]. Moreover, Asians, such as Japanese and Chinese, have a higher proportion of poor metabolites relative to Caucasian and African populations, therefore, strong gastric acid suppression can be obtained even with low doses of PPI [22]. However, even after adjusting for race, there was no PPI impact on OS in our study. Our unique study population is racially diverse which enhances the generalizability of the results.

Our study has several limitations, in particular, due to the retrospective nature of the analysis. Due to this research method, all data were extracted from patient charts, though every effort was made to limit errors at each step. Due to the nature of chart review data, it is also not certain that the patients listed as taking PPIs were compliant with the medications or were using the medication intermittently. Moreover, it is extremely hard to determine the intake duration accurately. In addition, the measured outcome in our study is OS rather than cancer-specific survival. Cancer-specific survival would be an important endpoint to study given the purported physiologic interaction between PPI use and CRC progression. Furthermore, all the patients in the study, after data refinement, underwent surgery, and this explains the relatively low percentage of patients with advanced stages (stages III and IV). On the other hand, our study has a large sample size, as well as a multi-racial population which is unique and helps in generalizing the results on the general population.

## Conclusions

In a large retrospective study of racially diverse CRC patients, our study did not demonstrate an association between PPI use and CRC OS. Future studies would ideally be prospective, collect data about PPI history prior to and after diagnosis of CRC, and include a measure of cancer-specific survival in the outcomes analysis. We would suggest that, until high-quality prospective data are available, oncologists and other physicians not stop PPIs that are otherwise clinically indicated.
